# Evaluation of Changes in Clinicopathological Features and Prognosis in Patients with Thyroid Cancer

**DOI:** 10.3390/jcm14051482

**Published:** 2025-02-23

**Authors:** Özlem Doğan, Melin Aydan Ahmed, Ömer Burak Ekinci, Anıl Yıldız, Izzet Dogan

**Affiliations:** 1 Department of Endocrinology, Health Sciences University, Haseki Training and Research Hospital, Istanbul 34265, Turkey; 2 Department of Medical Oncology, Institute of Oncology, Istanbul University, Istanbul 34193, Turkey; 3 Department of Medical Oncology, Health Sciences University, Prof. Dr. Cemil Tascioglu City Hospital, Istanbul 34284, Turkey; 4 Department of Medical Oncology, Health Sciences University, Basaksehir Cam and Sakura City Hospital, Istanbul 34480, Turkey; 5 Department of Medical Oncology, Acıbadem Healthcare Group, Istanbul 34140, Turkey

**Keywords:** thyroid cancer, SEER database, prognosis, clinicopathological features

## Abstract

**Background:** In this study, we evaluated the changes in clinicopathological features and prognosis in patients with thyroid cancer in the last two decades using the Surveillance, Epidemiology, and End Results Database (SEER) data. **Methods:** Data from the SEER-12 registry (1992–2021) were analyzed, focusing on patients diagnosed with malignant thyroid cancer between 2001 and 2020. The study population was divided into Cohort 1 (2001–2010) and Cohort 2 (2011–2020). Cohorts 1 and 2 were compared regarding clinicopathological features and prognosis. **Results:** The study included 94,892 patients diagnosed with thyroid cancer between 2001 and 2020, with 39,265 patients in Cohort 1 and 55,627 in Cohort 2. Compared to Cohort 1, in Cohort 2 showed a statistically significant increase in the proportion of patients aged 60+ (+4.2%), male patients (+2.1%), and cases of papillary cancer (+5.3%) and regional disease (+3.7%) (all *p* < 0.001). Although Cohort 2 demonstrated an 8% improvement in survival compared to Cohort 1, this result was not statistically significant (*p* = 0.057). Prognostic factors were identified, such as disease stage at diagnosis, age, gender, and origin. Among pathological subtypes, the patients with papillary + FV had the best prognosis (HR: 0.78), compared to patients in the other group, mainly comprising anaplastic tumors and sarcomas, which had the worst prognosis (HR: 9.61). **Conclusions:** In this large-scale study of thyroid cancer patients, we found significant differences between the two cohorts. In Cohort 2, the proportion of patients aged ≥60 years, male, and with papillary thyroid cancer was increased. We found that age, sex, origin, histopathological subtype, and stage at diagnosis were prognostic factors in patients with thyroid cancer. Also, we observed a trend toward improved survival in Cohort 2.

## 1. Introduction

Thyroid cancer is the most commonly diagnosed malignancy of the endocrine system, with an increasing global incidence over the past few decades [1]. It accounts for approximately 2% of all cancers and is predominantly diagnosed in women, with a notable age-related distribution, often affecting individuals between the ages of 20 and 55 [2]. The majority of thyroid cancers are differentiated, including papillary and follicular subtypes, which typically have a favorable prognosis. However, more aggressive forms, such as anaplastic and medullary thyroid cancers, are less common and are associated with poorer outcomes. Advances in early detection, imaging techniques, and improved surgical treatments contribute to an overall improvement in survival rates, yet regional and metastatic disease remains a significant concern [3].

Studies have identified various clinical and pathological factors that affect the prognosis of thyroid cancer patients. The main prognostic factors include age at diagnosis, gender, tumor size, lymph node involvement, extrathyroidal spread, and distant metastasis [4]. However, the most important prognostic factor is the tumor’s histological subtype. Papillary thyroid carcinoma (PTC) is generally associated with a better prognosis, while follicular, medullary, and anaplastic thyroid cancers (ATC) tend to have worse prognosis [5]. Studies in thyroid cancer also highlight the role of genetic mutations, such as those in the BRAF and RAS genes, which are linked to more aggressive disease and poorer survival outcomes [6]. Understanding prognostic factors is critical for tailoring individualized treatment plans and improving patient outcomes.

Today, the incidence of thyroid cancer has increased significantly, especially in small and localized PTCs, probably due to the increased use of neck ultrasonography and fine needle aspiration (FNA) [7]. This increased detection rate has led to earlier diagnosis, but it has also raised concerns about overtreatment. Economic, social, and demographic changes occurring in societies may lead to lifestyle changes in areas such as nutrition, smoking, alcohol use, and sports [8]. These changes necessitate a re-evaluation of prognostic factors and survival outcomes using large population-based databases. In this study, we evaluated the changes in clinicopathological and survival characteristics of thyroid cancer patients in the SEER database between 2001–2010 and 2011–2020.

## 2. Materials and Methods

### 2.1. Patients and Data Selection

This study was designed as a retrospective cohort study. The patients included in the study were obtained from the SEER Program of the National Cancer Institute (NCI), and the patient data were anonymized. The study was conducted in accordance with the declaration of Helsinki and good clinical practice guidelines. We obtained data for this study from the US NCI’s SEER-12 registry [November 2023 (1992–2021)]. SEER*Stat program version 8.4.4 was used to extract data. The patient population to be included in the study was determined by selecting Site recode ICD-O-3/WHO 2008 = ‘’thyroid” and Behavior code ICD-O-3 = ‘’malignant’‘ from the SEER*Stat program. Patients to be included in the study were restricted as Age recode with < 1-year olds = “20–24, 25–29, …, 80–84 and 85 years +’’ and Year of diagnosis= “2001, 2002, 2003, …, 2019, and 2020”. From the SEER*Stat program, patients’ Sex, Race recode, Origin recode, AYA site recode 2020 Revision, Histologic Type ICD-O-3, Combined Summary Stage (2004+), Summary stage 2000 (1998–2017), RX Summ--Scope Reg LN Sur (2003+), Radiation recode, Chemotherapy recode (yes, no/unk), and SEER cause-specific death classification data was saved.

The patient group was divided into two cohorts. Patients registered between 2001–2010 were considered Cohort 1, and patients registered between 2011–2020 were assigned to Cohort 2. Patients’ ages were divided into 20–39, 40–59, and 60+. Patients registered according to AYA site recode 2020 revision were recorded as “Other” except for papillary, papillary with follicular variant (FV), follicular, hurthle cell, and medullary. Surgery status, according to RX Summ-Surg Prim Site (1998+), and lymph node sampling status, according to RX Summ-Scope Reg LN Sur (2003+), were divided into three groups as “Yes, No and Unknown”. Radiation recode is divided into four groups as: “Radioisotopes, Radiotherapy (Implant and/or external), Radioisotopes+Radiotherapy, and None/Unknown”. While calculating the overall survival (OS) of the patients, “Survival months” were taken as the duration, and analysis was performed with patients coded “Dead (attributable to this cancer dx)” according to SEER cause-specific death classification. In addition, univariate and multivariate analyses were performed to determine the factors affecting OS.

### 2.2. Statistical Analysis

The SPSS 25.0 software (IBM, Armonk, NY, USA) and MedCalc statistical software version 23.0.9 (Ostend, Belgium) were used to perform the study statistics. The characteristics of patients in Cohorts 1 and 2 were compared using the Chi-square test. We used the Kaplan–Meier technique to analyze survival. Cox regression test was performed for univariate and multivariate analysis to evaluate the factors affecting survival in patients with thyroid cancer. Hazard ratios (HRs) and their 95% confidence intervals (CI) were estimated. Statistically significant variables from the univariate model have been included in the multivariate model. Chemotherapy, radiotherapy, radioisotope therapy, and surgical treatments were not included in multivariate analysis because they were correlated with tumor stage at diagnosis in patients with thyroid cancer. Statistical significance was accepted as a *p*-value < 0.05.

## 3. Results

### 3.1. Patient Characteristics and Treatment Modalities

A total of 94,892 patients were included in the study. The number of patients in Cohort 1 was 39,265, while the number in Cohort 2 was 55,627. The female/male ratio of patients included in the study was 3.01. The proportion of patients over the age of 60 in the study was determined to be 28.7%. The most common pathological subtypes were papillary, papillary with FV, and follicular types, respectively. Only 3.7% of patients were metastatic at the time of diagnosis, while the majority of patients presented with localized disease (58.4%). General characteristics and treatment characteristics of the patients are presented in Table 1.

### 3.2. Differences Between Cohort Groups

Cohort 1 and Cohort 2 were compared in terms of several parameters. In Cohort 2, the proportion of patients aged 60+ (+4.2%) and male patients (+2.1% increase) was detected to be increased (both *p* < 0.001). The origin rates were statistically significantly different between the two groups. When evaluated in terms of pathological subtype, there was a statistically significant increase of 5.3% in the proportion of patients with PTC (*p* < 0.001). There was also a 3.7% increase in the proportion of regional disease (*p* < 0.001). General and treatment-related differences in Cohort 2 compared to Cohort 1 are shown in Table 2.

### 3.3. Survival Outcomes and Prognosis

**Figure 1:**
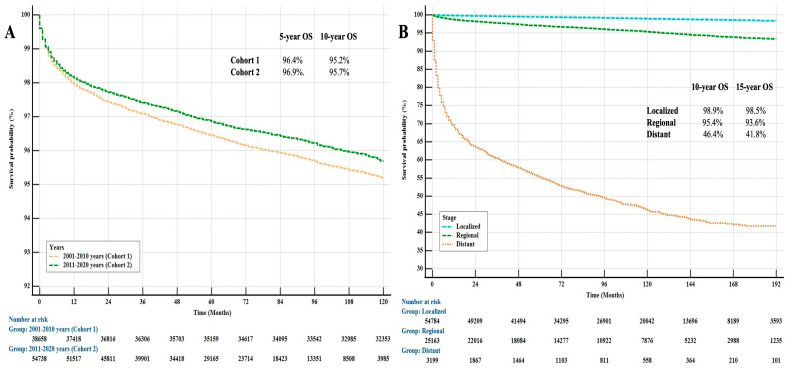
Kaplan–Meier Curves for OS by Cohorts (**A**) and Stages at Diagnosis (**B**) in Patients with Thyroid Cancer.

Of the patients included in the study, 3986 (4.2%) had died due to thyroid cancer. Univariate and multivariate analyses were performed to evaluate the factors affecting survival, and significant differences were observed between the Cohorts (Table 3). Although an 8% increase in survival was found in Cohort 2 compared to Cohort 1, this result remained below the statistical significance limit (*p* = 0.057) (Figure 1A). As expected, the disease stage at diagnosis was a prognostic factor (Figure 1B).

**Figure 2:**
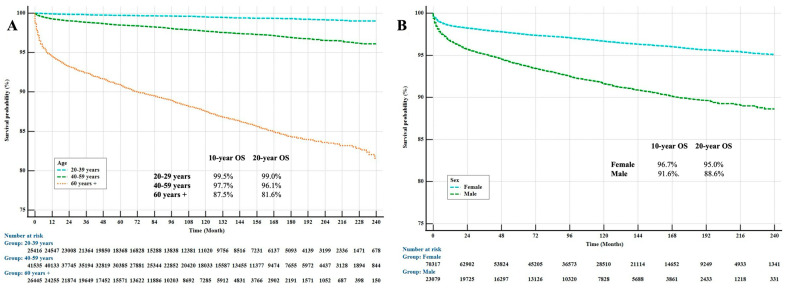
Kaplan–Meier Curves for OS by Age (**A**) and Sex (**B**) in Patients with Thyroid Cancer.

Age (95% CI, HR: 4.84) (Figure 2A) and gender (95% CI, HR: 1.42) (Figure 2B) were shown to be significant prognostic factors in patients with thyroid cancer. In addition, we showed that origin had a prognostic factor in patients with thyroid cancer (95% CI, HR: 0.79) in the multivariate analysis.

**Figure 3:**
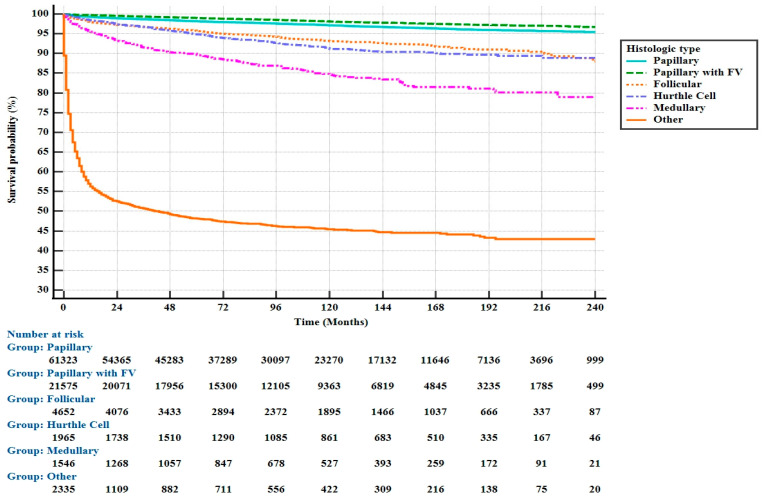
Kaplan–Meier Curves for OS by Pathologic Subgroups in Patients with Thyroid Cancer.

When evaluated in terms of pathological subtypes, the type with the best prognosis compared to the papillary type was papillary + FV (95% CI, HR: 0.78), and the type with the worst prognosis was the other group (95% CI, HR: 9.61) (Figure 3). The other group mainly included ATC and sarcomas.

## 4. Discussion

In this study, we compared the clinical features and prognoses of patients with thyroid cancer registered between 2001–2010 and 2011–2020 in the SEER database. Over the past several decades, advancements in early detection, treatment strategies, and molecular profiling have significantly influenced various cancers’ clinical presentation and prognosis. In particular, improvements in screening methods (such as mammography for breast cancer and colonoscopy for colorectal cancer) have contributed to earlier detection and, consequently, improved survival rates. In our study, comparing Cohort 1 and Cohort 2, we found that the proportion of patients aged 60+ and male patients tended to increase in recent years. Few studies in the literature have examined trends in thyroid cancer demographics with different findings regarding age and gender distributions. A study analyzing US data from 1976 to 2005 also observed that PTC incidence rates increased faster in women than in men, with the gender gap widening over time [9]. In particular, the female-to-male incidence ratio decreases with age, from over five in individuals aged 20–24 to approximately one in individuals aged 80 and over, showing a convergence in incidence rates between the sexes in older age groups. Consistent with our findings, a study conducted in the Balearic Islands between 2000 and 2020 reported that the mean age at diagnosis increased from 47.3 years in the previous decade to 52.1 years in the following decade [10]. These changes may reflect aging populations and improved diagnostic practices that detect thyroid cancer in older adults. However, gender trends remained consistent in this study, with proportionally similar thyroid cancer incidence in women compared to men throughout the study period.

One of the significant results we found in our study was the changes in the disease stages at diagnosis in the patients. Although diagnostic possibilities have increased over the years, we have shown that, contrary to expectations, there has been an increase in the proportion of patients in the regional stage and a decrease in the proportion of patients diagnosed with localized disease. A study conducted in Switzerland between 1998 and 2012 noted a significant increase in early-stage thyroid cancer diagnoses without an increase in advanced-stage cases, reinforcing the idea that less aggressive cancers are being overdiagnosed due to improved detection technologies and diagnostic practices [11]. This pattern contrasts with the decrease in rates of early-stage disease observed in our study and likely reflects differences in health systems or population characteristics. Another analysis noted that shifts in staging criteria, such as the adoption of the eighth edition of the TNM staging system, led to systematic downstaging of many cases of differentiated thyroid cancer [12]. This can be useful for interpreting trends over time, as changes in staging systems can affect the proportion of cases categorized as localized or regional. Direct comparisons of stage distributions are further complicated by the focus of modern staging systems on predicting mortality rather than recurrence.

We found a 5.3% increase in the frequency of PTC in the histopathological subgroup assessment in Cohort 2. Similarly, many studies have reported an increase in the frequency of PTC due to advances in diagnostic techniques such as high-resolution ultrasound and FNA biopsy. Similarly, this increase has been observed in various populations. For example, studies have shown that the incidence of PTC has more than doubled in certain regions, including South Korea, in recent decades, and this has been attributed primarily to increased screening efforts [13]. This trend has also been confirmed in other countries, with studies highlighting a significant rise in PTC diagnoses due to the more widespread use of imaging and pathological examinations [14]. Additionally, molecular alterations, such as those in the TERT promoter, have been shown to correlate with more aggressive PTC subtypes, further complicating the prognosis and treatment strategies [15].

In the survival analysis of patients with thyroid cancer, we determined that age, gender, origin, stage at diagnosis, and histopathological subtypes significantly affected survival statistically. The best prognostic group in histopathology was papillary + FV, while the worst was the other group, which mainly included ATC and sarcomas. Few studies in the literature have also examined prognostic factors affecting survival in thyroid cancer patients. A population-based study found that tumor differentiation status, age, and stage at diagnosis were strong predictors of survival, with increasing age being associated with lower relative survival for each histological type [16]. Similarly, another study reported that age greater than 60 years is associated with a worse prognosis in patients with PTC [17]. However, while race is a significant determinant in some studies, it appears to interact with other factors like age and stage rather than acting as an independent prognostic factor [18]. In the literature, there are studies on factors affecting overall survival in patients with thyroid cancer, as well as different studies examining factors affecting recurrence. In a recently published study evaluating factors predicting recurrence in patients with early-stage thyroid cancer, the presence of extrathyroid tumor extension and neck lymph node metastasis in patients with PTC were found to be statistically significant prognostic factors [19].

When comparing the cohorts in terms of prognosis, we found that, although the frequency of patients with regional-stage disease was increased in our study, there was a trend towards improved prognosis in patients with thyroid cancer in Cohort 2. There was an 8% survival improvement in patients in Cohort 2 compared with Cohort 1. This situation is thought to be possible due to the new drugs that have been used in cancer treatment in recent years. Several studies have examined the impact of new therapies on the prognosis of thyroid cancer, finding similar trends of survival improvement linked to novel therapies. In a recent study, it was found that combining targeted therapies and immunotherapy significantly improved overall survival in patients with ATC [20]. Additionally, studies on ATC, which is known to be aggressive, demonstrate significant survival improvements over the past two decades with personalized treatment regimens that include targeted therapies such as BRAF mutation inhibitors [21]. Similarly, in a study focused on differentiated thyroid cancer with lung metastases, it was indicated that radioiodine therapy (RAI) had a positive effect on survival, particularly in younger patients and those with fewer metastases [22].

This study has some limitations as it is a retrospective cohort study. It is difficult to demonstrate causal relationships in retrospective studies as they cannot account for all possible confounding variables since the results have already been reported. In addition, different studies or institutions may have different standards for the quality and consistency of data collection, which may have led to a heterogeneous collection of information. Retrospective studies using historical data may lead to a sample that is less representative of the current population, which may limit the generalizability of the results. In addition, The NCI SEER Dataset is a very comprehensive database containing data on cancer patients. This database was created with data from many different cancer centers [23]. The differences in diagnosis, treatment, and imaging methods among cancer centers are a limitation in obtaining standardized data. Also, there are deficiencies regarding patient treatment data in the NCI SEER Dataset [24]. This situation makes it difficult to perform more in-depth statistical analyses.

## 5. Conclusions

In this large-scale study of twenty years of data from patients with thyroid cancer, we investigated the main factors affecting survival outcomes and prognosis. In the study, we revealed significant differences between the two cohorts; we found an increase in the proportion of older patients, males, and PTC in Cohort 2. Though an increasing trend in survival rates was observed in Cohort 2, the results were not statistically significant. We found that age, sex, gender, origin, stage at diagnosis, and pathological subtype, including papillary + FV subtype, were critical prognostic indicators. The strengths of this study were its large sample size and the comprehensive examination of survival data across multiple variables, which increased the reliability and generalizability of the study. Identifying age, gender, and cancer subtype as essential factors for prognosis may facilitate clinical decision-making and guide more personalized treatment strategies for thyroid cancer patients. In addition, by demonstrating changes in patient characteristics and survival over time, this study sets a valuable precedent for future studies to improve patient outcomes and understand the evolving nature of thyroid cancer.

## Figures and Tables

**Table 1:** General characteristics of thyroid cancer patients in the SEER database included between 2001 and 2020.

		Number N: 94,892	%
**Age**	*20–39 years*	25,609	27.0
	*40–59 years*	41,993	44.3
	*60 years+*	27,290	28.7
**Sex**	*Female*	71,235	75.1
	*Male*	23,657	24.9
**Cohort**	*Cohort 1 (2001–2010 years)*	39,265	41.4
	*Cohort 2 (2011–2020 years)*	55,627	58.6
**Race Recode**	*White*	73,448	77.5
	*Asian or Pacific Islander*	14,181	14.9
	*Black*	5120	5.4
	*American Indian/Alaska Native*	981	1.0
	*Unknown*	1162	1.2
**Origin Recode**	*Spanish-Hispanic-Latino*	16,958	17.9
	*Non-Spanish-Hispanic-Latino*	77,934	82.1
**Histologic** **Subtypes**	*Papillary*	62,057	65.4
	*Papillary with FV*	21,775	22.9
	*Follicular*	4723	5.0
	*Hurthle cell carcinoma*	1979	2.1
	*Medullary*	1573	1.7
	*Other*	2785	2.9
**Stage at Diagnosis**	*Localized*	55,420	58.4
	*Regional*	25,303	26.7
	*Distant*	3504	3.7
	*Unknown*	10,665	11.2
**Surgery**	*Yes*	89,605	94.4
	*No*	5030	5.3
	*Unknown*	257	0.3
**Lymph** **Node Sampling**	*Yes*	46,400	48.9
	*No*	42,163	44.4
	*Unknown*	6329	6.7
**Radiotherapy and/or RAI**	*RAI*	38,615	40.7
	*RAI + Radiotherapy*	308	0.3
	*Radiotherapy*	2558	2.7
	*None/Unknown*	53,411	56.3
**Chemotherapy**	*None/Unknown*	93,867	98.9
	*Yes*	1025	1.1
**Thyroid Cancer-Related Death**	*Alive or Other Causes Dead*	90,906	95.8
	*Yes*	3986	4.2

**Table 2:** Comparison of General Characteristics of Patient Groups in Cohorts 1 and 2.

		Cohort 1		Cohort 2		*p*-Value
		N	%	N	%	
**Age**	*20–39 years*	10,905	27.8	14,704	26.4	***p* < 0.001**
	*40–59 years*	18,041	45.9	23,952	43.1	
	*60 years+*	10,319	26.3	16,971	30.5	
		39,265	100.0	55,627	100.0	
**Sex**	*Female*	29,946	76.3	41,289	74.2	***p* < 0.001**
	*Male*	9319	23.7	14,338	25.8	
		39,265	100.0	55,627	100.0	
**Race Recode**	*White*	31,244	80.2	42,204	77.1	***p* < 0.001**
	*Asian or Pacific Islander*	5368	13.7	8813	16.1	
	*Black*	2019	5.2	3101	5.6	
	*American Indian/Alaska Native*	348	0.9	633	1.2	
		38,979	100.0	54,751	100.0	
**Origin Recode**	*Spanish-Hispanic-Latino*	6132	15.6	10,826	19.5	***p* < 0.001**
	*Non-Spanish-Hispanic-Latino*	33,133	84.4	44,801	80.5	
		39,265	100.0	55,627	100.0	
**Histologic Subtypes**	*Papillary*	24,452	62.3	37,605	67.6	***p* < 0.001**
	*Papillary with FV*	9583	24.3	12,192	21.9	
	*Follicular*	2272	5.8	2451	4.4	
	*Hurthle cell*	1050	2.7	929	1.7	
	*Medullary*	691	1.8	882	1.6	
	*Other*	1217	3.1	1568	2.8	
		39,265	100.0	55,627	100.0	
**Stage at Diagnosis**	*Localized*	20,077	67.7	35,343	64.8	***p* < 0.001**
	*Regional*	8200	27.6	17,103	31.3	
	*Distant*	1391	4.7	2113	3.9	
		29,668	100.0	54,559	100.0	
**Surgery**	*Yes*	37,293	95.1	52,312	94.4	***p* < 0.001**
	*No*	1906	4.9	3124	5.6	
		39,199	100.0	55,436	100.0	
**Lymph Node Samplin**	*Yes*	15,742	47.4	30,658	55.4	***p* < 0.001**
	*No*	17,497	52.6	24,666	44.6	
		33,239	100.0	55,324	100.0	
**Radiotherapy** **and/or RAI**	*RAI*	18,443	92.5	20,172	93.6	***p* < 0.001**
	*RAI + Radiotherapy*	178	0.9	130	0.6	
	*Radiotherapy*	1311	6.6	1247	5.8	
		19,932	100.0	21,549	100.0	
**Chemotherapy**	*None/Unknown*	38,872	99.0	54,995	98.9	***p* = 0.047**
	*Yes*	393	1.0	632	1.1	
		39,265	100.0	55,627	100.0	
**Thyroid Cancer-Related Death**	*Alive or Other Causes*	37,082	94.4	53,824	96.8	***p* < 0.001**
	*Yes*	2183	5.6	1803	3.2	
		39,265	100.0	55,627	100.0	

**Table 3:** Univariate and multivariate analysis for factors affecting OS in thyroid cancer patients included in the SEER database between 2001 and 2020.

	Univariate Analysis		Multivariate Analysis	
	Hazard Ratio (CI 95%)	*p*-Value	Hazard Ratio (CI 95%)	*p*-Value
**Age**				
*20–39 years*	Reference		Reference	
*40–59 years*	4.54 (3.76–5.48)	**<0.001**	4.84 (3.85–6.08)	**<0.001**
*60 years+*	26.62 (22.22–31.89)	**<0.001**	16.12 (12.91–20.15)	**<0.001**
**Sex**				
*Female*	Reference		Reference	
*Male*	2.50 (2.35–2.67)	**<0.001**	1.42 (1.32–1.53)	**<0.001**
**Cohorts**				
*Cohort 1*	Reference		Reference	
*Cohort 2*	0.88 (0.82–0.94)	**<0.001**	0.92 (0.86–1.00)	0.057
**Race**				
*White*	Reference		Reference	
*Asian or Pacific Islander*	1.18 (1.08–1.28)	**<0.001**	1.04 (0.94–1.14)	0.41
*Black*	1.25 (1.10–1.42)	**0.001**	1.09 (0.94–1.26)	0.22
*American Indian/Alaska Native*	1.08 (0.79–1.48)	0.595	1.22 (0.86–1.73)	0.26
**Origin**				
*Spanish-Hispanic-Latino*	Reference		Reference	
*Non-Spanish-Hispanic-Latino*	0.88 (0.81–0.96)	**0.004**	0.79 (0.71–0.86)	**<0.001**
**Histopathological subtype**				
*Papillary*	Reference		Reference	
*Papillary with FV*	0.64 (0.57–0.72)	**<0.001**	0.78 (0.69–0.89)	**<0.001**
*Follicular*	2.41 (2.13–2.74)	**<0.001**	1.76 (1.52–2.05)	**<0.001**
*Hurthle Cell*	2.83 (2.39–3.36)	**<0.001**	2.43 (2.01–2.95)	**<0.001**
*Medullary*	5.58 (4.83–6.46)	**<0.001**	2.71 (2.30–3.20)	**<0.001**
*Other*	34.54 (32.08–37.18)	**<0.001**	9.61 (8.74–10.57)	**<0.001**
**Stage at diagnosis**				
*Localized*	Reference		Reference	
*Regional*	4.61 (4.13–5.14)	**<0.001**	4.89 (4.38–5.46)	**<0.001**
*Distant*	81.49 (73.62–90.21)	**<0.001**	29.54 (26.42–33.02)	**<0.001**

Multivariate analysis model *p*-value < 0.001.

## Data Availability

The datasets generated and/or analyzed during the current study are available in the Surveillance, Epidemiology, and End Results (SEER) Program repository, https://seer.cancer.gov/data-software/ (accessed on 15 December 2024)

## References

[1] Sung H., Ferlay J., Siegel R.L., Laversanne M., Soerjomataram I., Jemal A., Bray F. (2021). Global Cancer Statistics 2020: GLOBOCAN Estimates of Incidence and Mortality Worldwide for 36 Cancers in 185 Countries. CA Cancer J. Clin..

[2] Kitahara C.M., Sosa J.A. (2016). The changing incidence of thyroid cancer. Nat. Rev. Endocrinol..

[3] Haugen B.R., Alexander E.K., Bible K.C., Doherty G.M., Mandel S.J., Nikiforov Y.E., Pacini F., Randolph G.W., Sawka A.M., Schlumberger M. (2016). 2015 American Thyroid Association Management Guidelines for Adult Patients with Thyroid Nodules and Differentiated Thyroid Cancer: The American Thyroid Association Guidelines Task Force on Thyroid Nodules and Differentiated Thyroid Cancer. Thyroid.

[4] Glikson E., Alon E., Bedrin L., Talmi Y.P. (2017). Prognostic Factors in Differentiated Thyroid Cancer Revisited. Isr. Med. Assoc. J..

[5] Ulisse S., Baldini E., Lauro A., Pironi D., Tripodi D., Lori E., Ferent I.C., Amabile M.I., Catania A., Di Matteo F.M. (2021). Papillary Thyroid Cancer Prognosis: An Evolving Field. Cancers.

[6] Ahmadi S., Landa I. (2024). The prognostic power of gene mutations in thyroid cancer. Endocr. Connect..

[7] Davies L., Welch H.G. (2006). Increasing incidence of thyroid cancer in the United States, 1973–2002. JAMA.

[8] Rahelić V., Perković T., Romić L., Perković P., Klobučar S., Pavić E., Rahelić D. (2024). The Role of Behavioral Factors on Chronic Diseases—Practice and Knowledge Gaps. Healthcare.

[9] Kilfoy B.A., Devesa S.S., Ward M.H., Zhang Y., Rosenberg P.S., Holford T.R., Anderson W.F. (2009). Gender is an age-specific effect modifier for papillary cancers of the thyroid gland. Cancer Epidemiol. Biomark. Prev..

[10] Tofe S., Arguelles I., Forteza A., Alvarez C., Repetto A., Masmiquel L., Rodriguez I., Losada E., Sukunza N., Cabrer M. (2023). Age-standardized incidence, mortality rate, and trend changes of thyroid cancer in the Balearic Islands during the 2000–2020 period: A population-based study. Eur. Thyroid J..

[11] Jegerlehner S., Bulliard J.L., Aujesky D., Rodondi N., Germann S., Konzelmann I., Chiolero A., Group N.W. (2017). Overdiagnosis and overtreatment of thyroid cancer: A population-based temporal trend study. PLoS ONE.

[12] Lamartina L., Leboulleux S., Terroir M., Hartl D., Schlumberger M. (2019). An update on the management of low-risk differentiated thyroid cancer. Endocr. Relat. Cancer.

[13] Bouayed Abdelmoula N., Abdelmoula B., Masmoudi I., Aloulou S. (2024). Review Histopathological-molecular classifications of papillary thyroid cancers: Challenges in genetic practice settings. Biomed. Healthc. Res..

[14] Lam A.K. (2022). Papillary Thyroid Carcinoma: Current Position in Epidemiology, Genomics, and Classification. Papillary Thyroid Carcinoma.

[15] Steinberg E., Dimitstein O., Morand G.B., Forest V.I., da Silva S.D., Pusztaszeri M., Alohali S., Payne R.J. (2024). Clinical and Histopathological Features of Thyroid Cancer with TERT Promoter Molecular Alterations in Isolation Versus with Concurrent Molecular Alterations: A Multicenter Retrospective Study. Cancers.

[16] Gilliland F.D., Hunt W.C., Morris D.M., Key C.R. (1997). Prognostic factors for thyroid carcinoma. A population-based study of 15,698 cases from the Surveillance, Epidemiology and End Results (SEER) program 1973–1991. Cancer.

[17] Kauffmann R.M., Hamner J.B., Ituarte P.H.G., Yim J.H. (2018). Age greater than 60 years portends a worse prognosis in patients with papillary thyroid cancer: Should there be three age categories for staging?. BMC Cancer.

[18] Milano A.F. (2018). Thyroid Cancer: 20-Year Comparative Mortality and Survival Analysis of Six Thyroid Cancer Histologic Subtypes by Age, Sex, Race, Stage, Cohort Entry Time-Period and Disease Duration (SEER*Stat 8.3.2) A Systematic Review of 145,457 Cases for Diagnosis Years 1993–2013. J. Insur. Med..

[19] Marongiu A., Nuvoli S., De Vito A., Mura A., Vargiu S., Spanu A., Madeddu G. (2024). The Role of Risk Factors for the Progression of Patients with T1b-T2 Papillary Thyroid Carcinoma (PC) during Long-Term Follow-Up. J. Clin. Med..

[20] Cabanillas M.E., Dadu R., Ferrarotto R., Gule-Monroe M., Liu S., Fellman B., Williams M.D., Zafereo M., Wang J.R., Lu C. (2024). Anti-Programmed Death Ligand 1 Plus Targeted Therapy in Anaplastic Thyroid Carcinoma: A Nonrandomized Clinical Trial. JAMA Oncol..

[21] Maniakas A., Dadu R., Busaidy N.L., Wang J.R., Ferrarotto R., Lu C., Williams M.D., Gunn G.B., Hofmann M.C., Cote G. (2020). Evaluation of Overall Survival in Patients With Anaplastic Thyroid Carcinoma, 2000–2019. JAMA Oncol..

[22] Zhang S., Zhu M., Zhang H., Liu H., Fan X., Zhang J., Yu F. (2024). The Effect of Radioiodine Therapy on the Prognosis of Differentiated Thyroid Cancer with Lung Metastases. Biomedicines.

[23] About the SEER Registries. https://seer.cancer.gov/registries/.

[24] SEER Acknowledgment of Treatment Data Limitations. https://seer.cancer.gov/data-software/documentation/seerstat/nov2023/treatment-limitations-nov2023.html.

